# Predictive markers related to local and systemic inflammation in severe COVID-19-associated ARDS: a prospective single-center analysis

**DOI:** 10.1186/s12879-023-07980-z

**Published:** 2023-01-11

**Authors:** Jan Nikolaus Lieberum, Sandra Kaiser, Johannes Kalbhenn, Hartmut Bürkle, Nils Schallner

**Affiliations:** 1 Department of Anesthesiology and Critical Care Medicine, Medical Center, University of Freiburg, Freiburg, Germany; 2 Faculty of Medicine, University of Freiburg, Freiburg, Germany

**Keywords:** COVID-19, ICU, TLR3, IL-6, IL-8, Carboxyhemoglobin, Quality of Life, BAL, Outcome assessment

## Abstract

**Background:**

As the COVID-19 pandemic strains healthcare systems worldwide, finding predictive markers of severe courses remains urgent. Most research so far was limited to selective questions hindering general assumptions for short- and long-term outcome.

**Methods:**

In this prospective single-center biomarker study, 47 blood- and 21 bronchoalveolar lavage (BAL) samples were collected from 47 COVID-19 intensive care unit (ICU) patients upon admission. Expression of inflammatory markers toll-like receptor 3 (TLR3), heme oxygenase-1 (HO-1), interleukin (IL)-6, IL-8, leukocyte counts, procalcitonin (PCT) and carboxyhemoglobin (CO-Hb) was compared to clinical course. Clinical assessment comprised acute local organ damage, acute systemic damage, mortality and outcome after 6 months.

**Results:**

PCT correlated with acute systemic damage and was the best predictor for quality of life (QoL) after 6 months (r = − 0.4647, p = 0.0338). Systemic TLR3 negatively correlated with impaired lung function (ECMO/ECLS: r = − 0.3810, p = 0.0107) and neurological short- (RASS mean: r = 0.4474, p = 0.0023) and long-term outcome (mRS after 6 m: r = − 0.3184, p = 0.0352). Systemic IL-8 correlated with impaired lung function (ECMO/ECLS: r = 0.3784, p = 0.0161) and neurological involvement (RASS mean: r = − 0.5132, p = 0.0007). IL-6 in BAL correlated better to the clinical course than systemic IL-6. Using three multivariate regression models, we describe prediction models for local and systemic damage as well as QoL. CO-Hb mean and max were associated with higher mortality.

**Conclusions:**

Our predictive models using the combination of Charlson Comorbidity Index, sex, procalcitonin, systemic TLR3 expression and IL-6 and IL-8 in BAL were able to describe a broad range of clinically relevant outcomes in patients with severe COVID-19-associated ARDS. Using these models might proof useful in risk stratification and predicting disease course in the future.

*Trial registration* The trial was registered with the German Clinical Trials Register (Trial-ID DRKS00021522, registered 22/04/2020).

**Supplementary Information:**

The online version contains supplementary material available at 10.1186/s12879-023-07980-z.

## Introduction

The COVID-19 disease was first reported in late 2019 [1] and strains healthcare systems worldwide despite advancing vaccination programs with more than 600 million infections and 6.5 million deaths reported to WHO as of September 2022 [2]. In addition to many asymptomatic and mild courses, an infection can lead to clinically severe courses, resulting in acute lung failure (ARDS, acute respiratory distress syndrome), which has a high mortality specifically in COVID-19 [3].

Apart from getting a better understanding of the disease, finding biomarkers which can predict its course remains a crucial field of research, as clinical and epidemiological markers alone often do not reflect the clinical course [4]. Among the most discussed predictive markers are cytokines such as IL-6 (Gene ID: 3569) [4–6] and IL-8 (Gene ID: 3576) [4, 7, 8]. Among the further potential biomarkers are TLR 3 (Gene ID: 7098) which is responsible for recognizing viral double-stranded ribonucleic acid (dsRNA) [9, 10] and general markers of inflammation such as the leukocyte count [6, 10] and PCT [6, 11].

Another indicator for critical illness which is associated with increased mortality in patients is CO-Hb [12, 13], however its relevance in COVID-19 is still being discussed [14–16]. CO-Hb results from the binding of carbon monoxide with hemoglobin, with the predominant source of endogenous carbon monoxide being the degradation of heme by heme oxygenase. The inducible isoform HO-1 (Gene ID: 3162) was shown to be elevated in rats during ARDS [17], which makes a relevance of HO-1 and CO-Hb in COVID-19 likely.

Most of the predictive models published so far focused on selective questions such as mortality [4, 7], disease severity [11] or the need for mechanical ventilation [6] and/or included the heterogenous entire range of COVID-19 patients from mild to severe cases [4, 7, 11]. Thereby hindering general assumptions for relevant outcomes such as long-term functional capacity or disease related QoL.

Since treatment and care of COVID-19 patients requiring maximum-level ICU treatment is resource intensive and associated with a high rate of mortality [18], we focused on this specific subgroup of patients. Our aim was to get a better understanding of the inflammatory pathomechanisms of critical courses. Furthermore, we aimed at finding biomarkers that reflect the clinical and neurological short- and long-time outcome in severe COVID-19-associated ARDS, successfully building clusters of markers that enable prediction of disease course.

## Materials and methods

### Study design

**Fig. 1:**
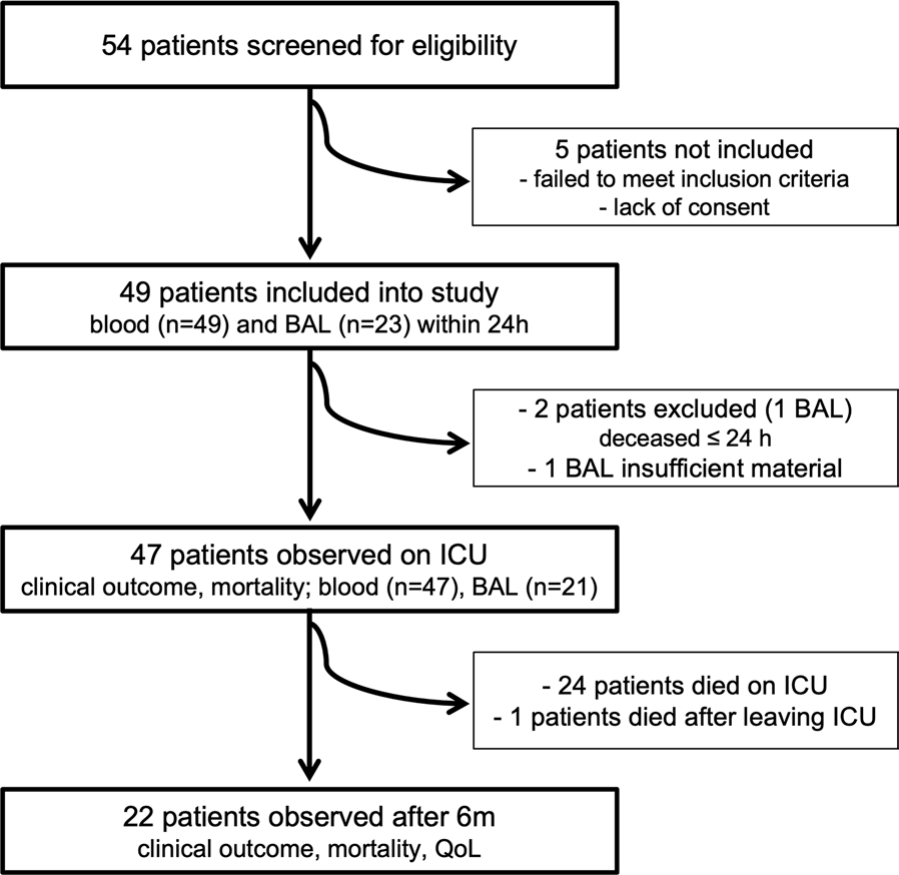
Schematic description of the study design. *BAL* bronchoalveolar lavage, *ICU* intensive care unit, *QoL* quality of life

This single-center prospective biomarker study aimed at observing possible correlations between markers reflecting the patients’ inflammatory status and ICU-relevant clinical outcomes. 47 patients with severe courses of COVID-19 admitted to the tertiary-level ICU and specialized ARDS treatment center of the Department of Anesthesiology and Critical Care, Medical Center, Faculty of Medicine—University of Freiburg (Germany) between April 2020 and April 2021 were included (Fig. 1). For inclusion and exclusion criteria see Additional file 1: Methods. The study protocol was approved by the Institutional Ethics Review Board of the University of Freiburg (Protocol No. 225/20) and informed consent was provided. The trial was registered with the German Clinical Trials Register (Trial-ID DRKS00021522, registered 22/04/2020). All procedures performed in the study were in accordance with the ethical standards of the institutional research committee and with the 1964 Helsinki Declaration. Data reporting adheres to the STROBE guidelines.

A secondary analysis to this study investigated the cost-utility of ICU treatment of COVID-19 patients [19].

### Sample collection and analysis

BAL and blood samples were collected within 24 h after admission to ICU. BAL samples were obtained as aliquots from diagnostic BALs upon ICU admission. If no BAL was gathered within 24 h after admission, no BAL sample was included into our study.

Blood samples were used to collect serum and leukocyte RNA. BAL supernatant was separated and RNA isolated from the cell pellet. Procedures of RNA isolation and transcription into complementary desoxyribonucleic acid (cDNA) are described in the Additional file 1: Methods.

Real-time polymerase chain reaction (PCR) with nucleic acid stain was used for semi-quantification of cDNA. Using primers (primer sequences s. Additional file 1: Methods) for TLR3, HO-1 and ribosomal protein L13a (Rpl13a) we analyzed the gene expression in blood using −ΔΔCT method with Rpl13a as reference and five healthy controls (mean age 33.2 years; 40% male). Gene expression in BAL was analyzed using −ΔCT method as there were no healthy controls out of ethical reasons. Comparison to expression in blood was also done using −ΔCT method.

IL-6 and IL-8 cytokine secretion in 40 serum and 19 BAL supernatants were measured via flow cytometry using magnetic beads (Human Inflammatory Cytokine Cytometric Bead Array, BD#551811). The control population for blood samples consisted of four healthy controls (mean age 34.5 years, 50% male).

Out of ethical reasons, controls were recruited from volunteers in our lab and blood samples acquired from venous catheters instead of recruiting age- and sex-matched patients suffering from different diseases. The main reason for including controls was of technical nature to establish a baseline and to be able to report qPCR results using the −ΔΔCT method where possible.

During Sample analysis, investigators were blinded to the clinical course.

We used the first leukocyte (1st Leukos) and the first procalcitonin (1st PCT) value from the routine laboratory done in ICU. Except for one PCT measurement performed on day 5, all measurements were performed within 24 h after admission to ICU. For mean (Leukos mean) and maximum (Leukos max) leukocyte values, initial measurement at admission and one measurement per day -to a maximum of 14 days- were included. CO-Hb values were taken from blood gas analyses done in ICU.

Comorbidities and age were accounted for using the Charlson Comorbidity Index (CCI).

### Clinical assessment

The clinical assessment focused on four different questions: (1) acute local damage in lung and kidney, (2) acute systemic damage expressed by systemic inflammation, thromboembolic events and neurological involvement, (3) mortality and (4) neurological functional capacity and QoL after 6 months.

For local lung damage we used five surrogate markers: mean peak ventilation pressure (Ppeak), minimal and mean Horovitz index, need for nitric oxide (NO) supplementation and need for extracorporeal membrane oxygenation (ECMO)/extracorporeal life support (ECLS). Local kidney damage was assessed by occurrence of acute kidney injury (AKI) defined by the KDIGO criteria and need for dialysis.

Systemic inflammation was measured by the mean and maximum leukocyte values. Thromboembolic events, defined as segmental or subsegmental lung embolism, intracranial thromboembolism, thrombi in a bigger vessel such as the jugular veins or radial arteria or mesenterial ischemia, were confirmed by radiological imaging. In one case a pulmonary thromboembolic event was assumed when the clinical course immediately before death with sudden deterioration in oxygenation accompanied by right ventricular decompensation detected using echocardiography made it highly likely. Neurological involvement was assessed via minimal and mean Richmond Agitation Sedation Scale (RASS), need for sedation and modified Rankin scale (mRS) at admission, after 14 days, at discharge and after 6 months. Additionally, QoL after 6 months was measured using the 5-level EQ-5D questionnaire introduced by the EuroQol Group (version validated for German population) [20].

Except for mRS at discharge and mortality, all assessment in ICU was limited to the first 14 days to focus on the acute disease course directly caused by COVID-19 instead of complications caused by prolonged stay in ICU such as superinfections or ICU-acquired weakness. Duration of need for NO supplementation, ECMO/ECLS support or sedation were normalized to the length of stay in ICU to account for patients who deceased/were discharged before day 14. Ppeak and Horovitz mean values during NO supplementation or ECMO/ECLS were not included to avoid false low/high values.

### Statistical analysis

Data analysis was performed using GraphPad Prism 9 software. Two groups were compared using two-tailed Mann–Whitney test or Wilcoxon matched-pairs signed rank test. Results are presented as means (+ 95% CI). For correlation analyses normality was tested with the Anderson–Darling test and subsequently either Spearman nonparametrical or Pearson parametrical correlation was used. Contingency analyses were done using Chi-squared and Fisher’s exact test.

First assessment for a potential use as predictive marker was done via receiver operating characteristic (ROC) using Wilson/Brown method. The relationship and possible interactions between biomarkers and clinical outcome (dichotomization according to Additional file 1: Methods) was explored using multiple logistic regression with CCI and sex always being included into the models. For comparisons of models, Akaike’s Information Criterion, Tjur’s R squared and Hosmer–Lemeshow hypothesis test were used. Our aim was to find either one common or separate models to describe local damage in lung and kidney, systemic damage, mortality and quality of life after 6 months.

An a priori power analysis (Chi-squared test contingency table; Inflammation high/low vs. mRS high/low; expected effect size Cohen’s w = 0.5; α = 0.05; β = 0.9) indicated a minimum sample size of n = 43.

Patients missing information for a certain analysis were not included into the specific analysis. p < 0.05 was considered statistically significant.

## Results

### Gender distribution of patients differed from the expectation

Patient characteristics and previous history before admission to ICU are summarized in Table 1. A total of 49 patients were initially included into the study with two being excluded when the therapeutic goal was changed to palliative care within 24 h after admission. 22 patients survived until the 6 months follow-up.

**Table 1:** Patient characteristics and previous history

**a** Patient characteristics	
Sex, n (%)	
Male	37 (78.7)
Female	10 (21.3)
Age (years)	
Mean	57.7
Median	59
IQR	11
Mortality, n (%)	
In ICU	24 (51.1)
After 6 months	25 (53.2)
Charlson Comorbidity Index	
Mean	2.3
Median	2
IQR	2
LOS ICU all (d)	
Mean	22.5
Median	17
IQR	21
LOS ICU survivors (d)	
Mean	30.5
Median	32
IQR	22.5
**b** previous history	
Onset of symptoms (d)	
Mean	13.3
Median	12
IQR	8
Days since first positive PCR on SARS-CoV-2	
Mean	9.5
Median	7
IQR	12
Days in hospital (including other ICU)	
Mean	6.9
Median	6
IQR	9

**a** Patient characteristics at admission and **b** previous history before admission to ICU. *IQR* interquartile range, *ICU* intensive care unit, *LOS* length of stay

In line with previous studies showing male sex as risk factor for disease progression [21], sex distribution in the study population compared to general society was significantly shifted towards more male patients [22] (p = 0.0049). Mean age was 57 (IQR 11) years. ICU and overall mortality were reported with 51 and 53%, respectively. The mean Charlson Comorbidity Index (CCI) was 2. A more detailed list of the patients’ comorbidities can be found in Additional file 1: Table S1. Chronic lung diseases and bronchial asthma did not influence biomarker expression in BAL, tumors and lymphoma did not influence biomarker expression in blood (ROC analyses, all p > 0.05). Patients had a mean length of ICU stay (LOS ICU) of 22.5 days overall and 30.5 days in the survivor subgroup. The average time of symptom onset to admission to the study ICU was 13 days with a mean time lag between the first positive PCR on SARS-CoV-2 and admission being 9.5 days. Patients had been hospitalized in normal wards and lower-level intermediate care units for an average of 7 days prior to specialized ICU admission. Age had no influence on mortality (p > 0.9999) or LOS ICU (p = 0.8769). Bacterial superinfection was defined as positive bacterial sampling with therapeutic consequences. 21.3% of the patients met these criteria at admission, 70.2% within the first 14 days after admission and 83.0% did ever meet them, including those sufficiently treated before admission to our ICU. Neither of the three time points showed influence on mortality in Chi-squared- or on QoL in ROC-analyses (all p > 0.05).

### First leukocyte value correlated with systemic inflammation and reduced lung function

Descriptive statistics for all biomarker measurements can be seen in Additional file 1: Table S2.

**Fig. 2:**
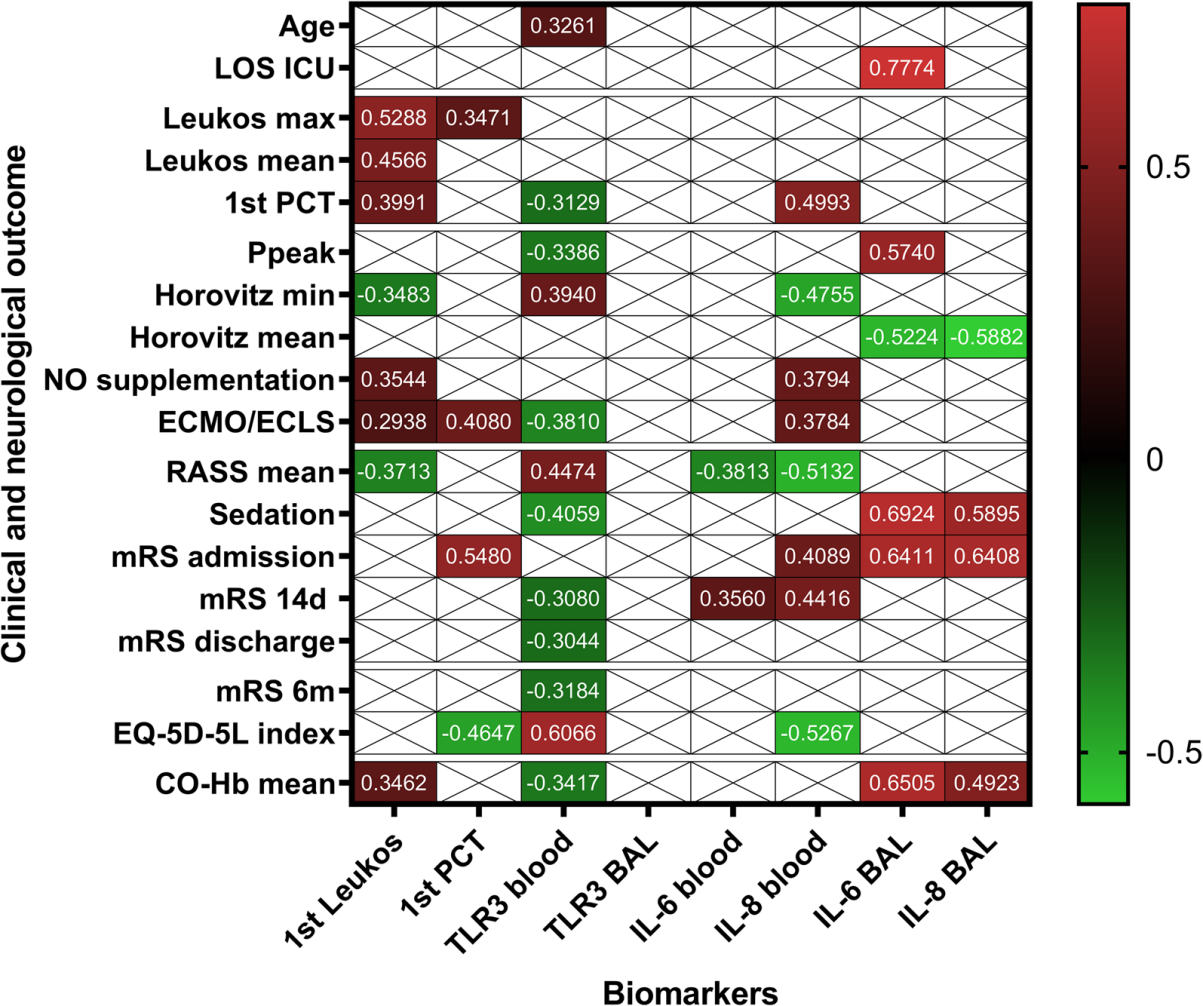
Heat map showing correlations of biomarkers vs. clinical and neurological short- and long-time outcome. Only r-values for significant correlations are shown. Depending on whether data showed normal distribution, Spearman nonparametrical or Pearson parametrical test was used. Cells with “X” did not reach significance. 1st PCT vs. 1st PCT not shown. For p-values, n and parameter statistics in multivariate analyses see Table 2. Correlations are visualised in Fig. 3. *LOS ICU* length of stay in intensive care unit, *Ppeak* mean peak ventilation pressure, *NO* nitric oxide, *ECMO/ECLS* extracorporeal membrane oxygenation/extracorporeal life support, *RASS* Richmond Agitation-Sedation Scale, *mRS* modified Rankin scale, *CO-Hb* carboxyhaemoglobin, *BAL* bronchoalveolar lavage

1st Leukos positively correlated with Leukos max, Leukos mean and 1st PCT (Fig. 2). The mean number of regarded leukocyte measurements per patient was 13.02 (median 15, IQR 2). Maximum levels occurred with a mean time lag of 6.04 days after admission to our ICU (median 6, IQR 7).

**Fig. 3:**
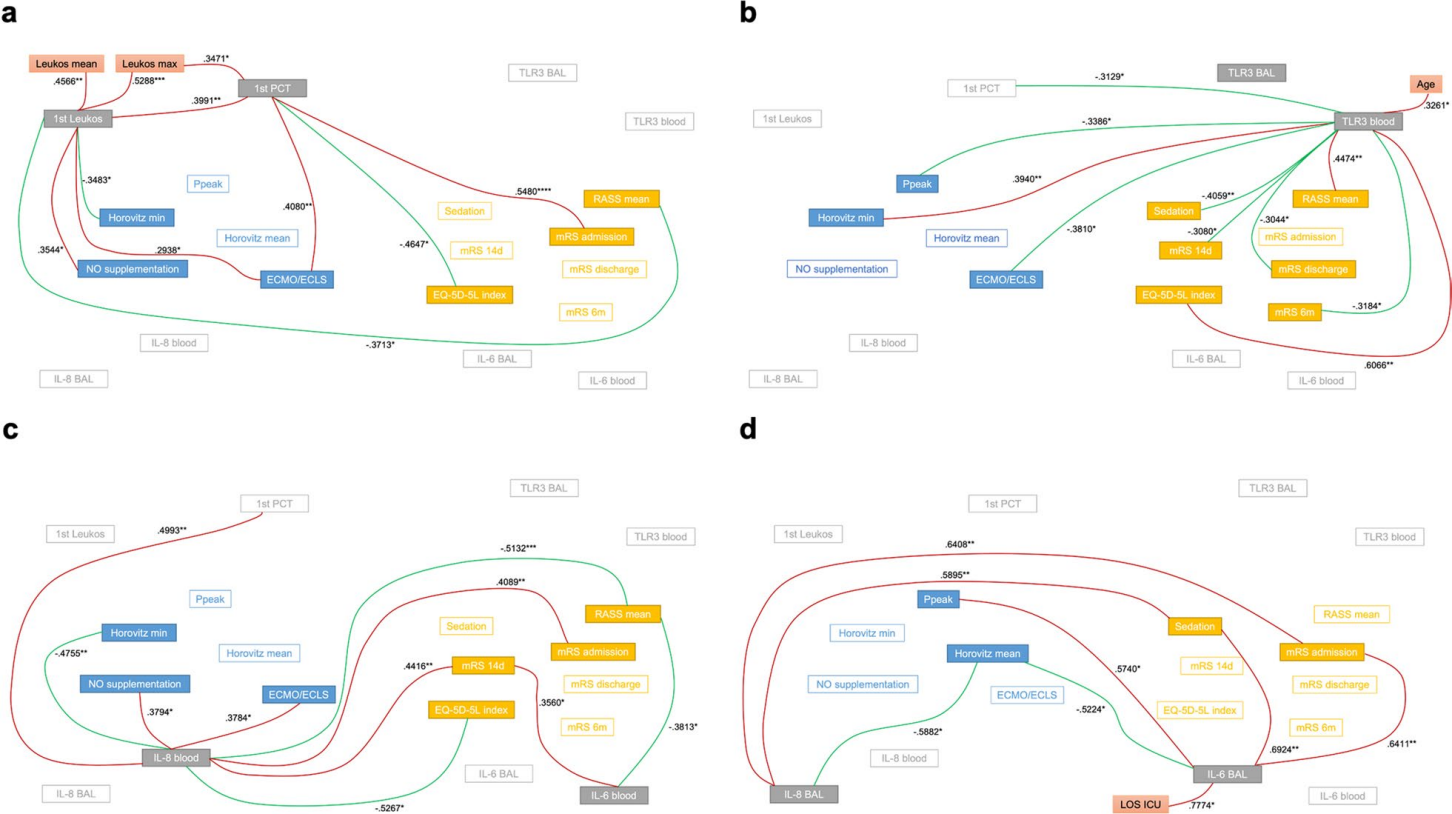
Network graphics showing correlations of biomarkers vs. outcome. **a** 1st Leukos and 1st PCT vs. outcome. **b** TLR3 in blood and BAL vs. outcome. **c** Cytokine secretion in blood vs. outcome. **d** Cytokine secretion in BAL vs. outcome. Color code: grey boxes, biomarker, blue boxes, parameter for lung impairment, yellow boxes, parameter for neurological involvement and QoL, red arrow, positive correlation, green arrow, negative correlation. *p < 0.05, **p < 0.01, ***p < 0.001, ****p < 0.0001. *LOS ICU* length of stay in intensive care unit, *Ppeak* mean peak ventilation pressure, *NO* nitric oxide, *ECMO/ECLS* extracorporeal membrane oxygenation/extracorporeal life support, *RASS* Richmond Agitation-Sedation Scale, *mRS* modified Rankin scale, *BAL* bronchoalveolar lavage

A high 1st Leukos correlated with a lower minimum Horovitz index (Horovitz min), more NO supplementation and more ECMO/ECLS support (Fig. 2). It also showed a negative correlation to the mean RASS score (Fig. 2), which remained statistically significant in a multivariate analysis with CCI and sex (Table 2). Statistics are summarized in Table 2. Correlations are visualized in Fig. 3A.

**Table 2:** Correlations of biomarkers vs. clinical and neurological short- and long-time outcome

Biomarker	Outcome	r-value	p-value	Multivariate analysis	n =
1st Leukos	Leukos max	0.5288	0.0001	N/A	47
	Leukos mean	0.4566	0.0013	N/A	47
	1st PCT	0.3991	0.0066	N/A	45
	Horovitz min	− 0.3483	0.0164	ns	47
	NO supplementation	0.3544	0.0145	N/A	47
	ECMO/ECLS	0.2938	0.0450	ns	47
	RASS mean	− 0.3713	0.0102	0.0450	47
1st PCT	Leukos max	0.3471	0.0195	N/A	45
	ECMO/ECLS	0.4080	0.0054	0.0162	45
	mRS admission	0.5480	< 0.0001	N/A	45
	EQ-5D-5L index	− 0.4647	0.0338	0.0138, Intercept* (0.0203)	21
TLR3 blood	Age	0.3261	0.0308	N/A	44
	1st PCT	− 0.3129	0.0436	N/A	42
	Ppeak	− 0.3386	0.0376	N/A	38
	Horovitz min	0.3940	0.0081	ns	44
	ECMO/ECLS	− 0.3810	0.0107	ns	44
	RASS mean	0.4474	0.0023	0.0159	44
	Sedation	− 0.4059	0.0063	0.0055	44
	mRS 14d	− 0.3080	0.0445	N/A	43
	mRS discharge	− 0.3044	0.0445	N/A	44
	mRS 6 m	− 0.3184	0.0352	0.0114	44
	EQ-5D-5L index	0.6066	0.0059	N/A	19
IL-6 blood	RASS mean	− 0.3813	0.0152	ns	40
	mRS 14d	0.3560	0.0261	N/A	39
IL-8 blood	1st PCT	0.4993	0.0012	N/A	39
	Horovitz min	− 0.4755	0.0019	ns	40
	NO supplementation	0.3794	0.0158	N/A	40
	ECMO/ECLS	0.3784	0.0161	ns	40
	RASS mean	− 0.5132	0.0007	ns	40
	mRS admission	0.4089	0.0088	N/A	40
	mRS 14d	0.4416	0.0049	N/A	39
	EQ-5D-5L index	− 0.5267	0.0142	Intercept* (0.0276)	21
IL-6 BAL	LOS ICU	0.7774	0.0104	N/A	10
	Ppeak	0.5740	0.0220	N/A	16
	Horovitz mean	− 0.5224	0.0399	ns	16
	Sedation	0.6924	0.0010	ns	19
	mRS admission	0.6411	0.0031	N/A	19
IL-8 BAL	Horovitz mean	− 0.5882	0.0185	ns	16
	Sedation	0.5895	0.0079	ns	19
	mRS admission	0.6408	0.0031	N/A	19

Shown are those correlations which reached significance. Depending on whether data showed normal distribution, Spearman nonparametrical or Pearson parametrical test was used for correlation testing. Akaike’s Information Criterion, Tjur’s R squared and Hosmer–Lemeshow hypothesis test were used for multivariate analyses. Multivariate analyses were composed of the respective biomarker, CCI and sex. Outcome parameters with a more descriptive than predictive value (when assessed at a similar time to sample acquisition, for example mRS at admission) were not tested in these models to avoid unnecessary tests. Similarly, markers with less clinical importance than other markers (for example Ppeak vs. Horovitz index) were not included into multivariate analyses. N/A, not tested or could not compute model. *p < 0.05. *LOS ICU* length of stay in intensive care unit, *Ppeak* mean peak ventilation pressure, *NO* nitric oxide, *ECMO/ECLS* extracorporeal membrane oxygenation/extracorporeal life support, *RASS* Richmond Agitation-Sedation Scale, *mRS* modified Rankin scale, *BAL* bronchoalveolar lavage

### First procalcitonin value correlated with short-time damage and quality of life after 6 months

There was no significant influence of bacterial superinfection at admission on 1st PCT (p > 0.05). 1st PCT positively correlated with Leukos max, ECMO/ECLS support, mRS at admission and negatively with EQ-5D-5L index after 6 months (Fig. 2). ECMO/ECLS support and EQ-5D-5L index remained statistically significant in multivariate analyses with CCI and sex. Statistics are summarized in Table 2. Correlations are visualized in Fig. 3A.

### Systemic TLR3 negatively correlated with reduced lung function and neurological short- and long-time damage

TLR3 expression in blood leukocytes showed no difference between healthy controls and COVID-19 patients (p = 0.8610). Systemic TLR3 positively correlated with age and negatively with 1st PCT (Fig. 2).

When compared to markers for impaired lung function, systemic TLR3 showed a positive correlation to Horovitz min and negative correlations to Ppeak and ECMO/ECLS support (Fig. 2). In multivariate analyses with CCI and sex, ECMO/ECLS narrowly missed significance (p = 0.0507).

For neurological short-time impairment, systemic TLR3 positively correlated to the mean RASS score while negatively correlating to sedation in ICU, mRS after 14 days and at discharge (Fig. 2). After 6 months, systemic TLR3 expression still showed a negative correlation to the mRS score and additionally, a positive correlation to the EQ-5D-5L index (Fig. 2). In multivariate analyses with CCI and sex, RASS mean, the required sedation and mRS after 6 months remained statistically significant (Table 2). TLR3 in BAL showed no significant correlations with outcome. Statistics are summarized in Table 2. Correlations are visualized in Fig. 3B.

### Systemic cytokine secretion correlated with reduced lung function and neurological impairment

IL-6 in serum showed a negative correlation to the mean RASS score and a positive correlation to the mRS score after 14 days (Fig. 2).

Systemic IL-8 secretion was higher in COVID-19 patients than in healthy controls (p < 0.0001). High systemic IL-8 correlated with a higher 1st PCT (Fig. 2). It also correlated with a lower Horovitz min, more NO supplementation as well as more ECMO/ECLS support (Fig. 2).

Systemic IL-8 showed a significant correlation with the mRS on admission and mRS after 14 days (Fig. 2) but not with the mRS at discharge or mRS after 6 months. It furthermore negatively correlated to the mean RASS score and the EQ-5D-5L index after 6 months (Fig. 2). In a multivariate analysis with CCI and sex for EQ-5D-5L index, the intercept but not systemic IL-8 achieved statistical significance (Table 2).

A separate analysis for correlation between systemic IL-8 and neutrophile concentration in blood showed a correlation to the first (r = 0.3698, p = 0.0288) but not mean and maximum neutrophile counts. Statistics are summarized in Table 2. Correlations are visualized in Fig. 3C.

### IL-6 and IL-8 in BAL correlated with lung and neurological damage as well as length of ICU stay

A higher IL-6 value in the BAL correlated with a longer LOS ICU for survivors, a higher Ppeak but a lower mean Horovitz index (Horovitz mean) (Fig. 2). Furthermore, IL-6 in the BAL positively correlated with mRS at admission and the required length of sedation (Fig. 2).

IL-8 in the BAL negatively correlated with Horovitz mean and positively with mRS on admission as well as the length of sedation (Fig. 2). IL-8 in BAL did not correlate to neutrophile counts. Statistics are summarized in Table 2. Correlations are visualized in Fig. 3D.

Correlations between biomarkers can be seen in Additional file 1: Figure S1. For the markers discussed so far, there was no correlation between expression in BAL and blood with higher levels of TLR3 and IL-8 in BAL.

First assessment of predictive potential was done using ROC analyses as shown in Additional file 1: Table S3. Mainly ECMO/ECLS could be predicted by a single marker.

### CCI, sex, 1st PCT and IL-8 BAL predict local damage in lung and kidney

A model consisting of intercept, CCI, sex, 1st PCT and IL-8 secretion in BAL was used in a multivariate analysis to describe the local damage in lung and kidney (Table 3).

**Table 3:** Multiple logistic regression models for local and systemic damage as well as quality of life

		AUC	PPP	NPP	Tjur‘s R2	n =	Variable	CCI	Sex [w]	1st PCT	IL-8 BAL
Model local damage	Horovitz min	**0.9091**	100.00	80.00	**0.5754**	19	OR	0.8183	7.423	593.8	0.9998
	> 72 mmHg = “negative”	95%-CI: 0.7668 to 1.000					95%CI	0.1032 to 2.989	0.2649 to 645.5	3.385 to 4.01E + 06	0.9991 to 1.000
		**p = 0.0030**					p	0.7941	0.2670	0.0601	0.3881
	Horovitz mean	0.7460	83.33	80.00	0.2456	16	OR	1.493	2.532	1.468	1.000
	> 150 mmHg = “negative”	95%-CI: 0.4774 to 1.000					95%CI	0.5036 to 4.709	0.1524 to 47.53	0.1687 to 8.105	1.000 to 1.001
		p = 0.1009					p	0.4392	0.5048	0.6477	0.1512
	ECMO/ECLS	**0.9659**	100.00	91.67	**0.7312**	19	OR	0.3400	0.07332	57.054	0.9989
	No ECMO/ECLS = “negative”	95%-CI: 0.8889 to 1.000					95%CI	0.01735 to 1.634	1.74E-07 to 34.45	8.282 to 6.22E + 13	0.9968 to 0.9998
		**p = 0.0007**					p	0.3368	0.5010	0.0787	0.0914
	Dialysis	**0.9889**	100.0	90.91	**0.8074**	19	OR	1.49E-17	4.00E + 20	77.444	1.004
	No Dialysis = “negative”	95%-CI: 0.9538 to 1.000					95%CI	6.21E-76 to 0.07919	1.060 to 3.49E + 81	2.082 to 2.29E + 18	0.9999 to 1.015
		**p = 0.0003**					p	0.3276	0.3572	0.3261	0.3611

		AUC	PPP	NPP	Tjur‘s R2	n =	variable	CCI	Sex [w]	TLR3 blood	IL-6 BAL
Model systemic damage	RASS mean	**0.9167**	87.50	77.78	**0.5197**	17	OR	1.146	1.650	0.1243	0.9997
	> − 4 = “negative”	95%-CI: 0.7822 to 1.000					95%CI	0.1782 to 8.219	0.01546 to 339.3	0.005356 to 0.6342	0.9970 to 1.001
		**p = 0.0039**					p	0.8797	0.8306	0.0693	0.6077
	Sedation	**0.9722**	100.0	88.89	**0.7544**	17	OR	2.700	2.07E-05	0.02232	1.007
	< 0.5 = “negative”	95%-CI: 0.9034 to 1.000					95%CI	3.286 to 5.59E + 16	2.28E-20 to 0.3901	1.61E-10 to 0.7858	1.001 to 1.028
		**p = 0.0011**					p	0.2463	0.2338	0.2972	0.1879
	mRS 6 m	**0.9429**	90.00	85.71	**0.6199**	17	OR	41.05	0.003212	0.008457	0.9987
	< 2 = “negative”	95%-CI: 0.8363 to 1.000					95%CI	1.712 to 1.38E + 06	2.45E-12 to 2.016	5.20E-09 to 0.3103	0.9952 to 0.9999
		**p = 0.0025**					p	0.1554	0.2462	0.1396	0.1540
	Thromboembolic events	**0.7917**	80.00	85.71	0.2705	17	OR	0.8522	0.6872	1.449	0.9988
	No event = “negative”	95%-CI: 0.5403 to 1.000					95%CI	0.1786 to 3.677	0.01146 to 23.33	0.5591 to 4.175	0.9967 to 1.000
		**p = 0.0433**					p	0.8270	0.8360	0.4452	0.1840
	Mortality	**0.9444**	87.50	77.78	**0.5711**	17	OR	28.99	0.04563	0.05985	0.9990
	Not deceased = “negative”	95%-CI: 0.8406 to 1.000					95%CI	1.951 to 4733	2.76E-05 to 4.201	5.79E-04 to 0.4601	0.9974 to 1.000
		**p = 0.0021**					p	0.0651	0.2416	0.0710	0.0895

		AUC	PPP	NPP	Tjur‘s R2	n =	Variable	CCI	Sex [w]	1st PCT
QoL 6 m	EQ-5D-5L index	**0.8942**	87.50	92.31	**0.5371**	21	OR	2.813	8.630	**1.291**
	> 0.7 = “negative”	95%-CI: 0.7393 to 1.000					95%CI	1.080 to 11.45	0.4123 to 384.3	1.086 to 1.672
		**p = 0.0030**					p	0.0661	0.1893	**0.0138**

Local damage $\sim$ Intercept + CCI + Sex [w] + 1st PCT + IL-8 BAL. Dependent variables for local damage: Horovitz min > 72 mmHg [favorable] vs. < 72 mmHg [unfavorable], Horovitz mean > 150 mmHg [favorable] vs. < 150 mmHg [unfavorable], ECMO/ECLS support yes/no, dialysis yes/no. [unfavorable] = “positive” outcome

Systemic damage $\sim$ Intercept + CCI + Sex [w] + TLR3 blood + IL-6 BAL. Dependent variables for systemic damage: RASS mean > − 4 [favorable] vs. RASS mean < − 4 [unfavorable], need for sedation < 0.5 [favorable] vs. > 0.5 [unfavorable], mRS after six months [mRS 6 m] 0–2 [favorable] vs. mRS 6 m 3–6 [unfavorable], thromboembolic events yes/no, mortality yes/no. [unfavorable] = “positive” outcome

QoL $\sim$ Intercept + CCI + Sex [w] + 1st PCT. EQ-5D-5L index > 0.7 [favorable] vs. < 0.7 [unfavorable]. [unfavorable] = “positive” outcome

Comparison method: Akaike’s Information Criterion, Tjur’s R squared, Hosmer–Lemeshow hypothesis test. Intercept not shown. Statistically significant AUC or OR as well as Tjur’s R^2^ > 0.5 are highlighted in bold. *RASS* Richmond Agitation-Sedation Scale, *mRS* modified Rankin Scale, *AUC* area under the curve, *CI* confidence interval, *PPP* positive predictive power, *NPP* negative predictive power, *OR* odds ratio

**Fig. 4:**
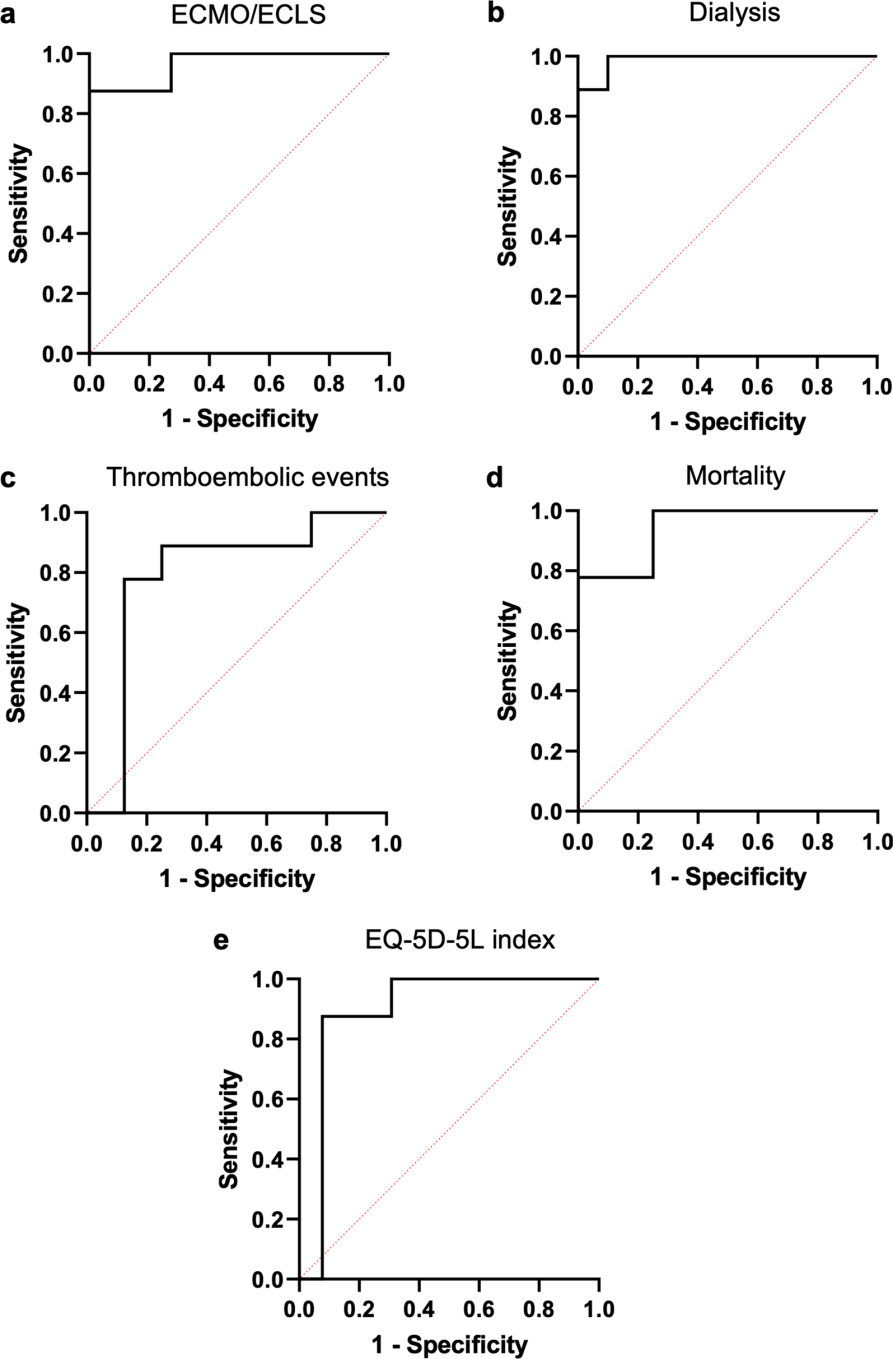
Multiple logistic regression models for local and systemic damage as well as quality of life. Model for local damage: **a** need for ECMO/ECLS and **b** need for dialysis. Model for systemic damage: **c** occurrence of thromboembolic events and **d** mortality. Model for quality of life: **e** EQ-5D-5L index. Comparison method: Akaike’s Information Criterion, Tjur’s R squared, Hosmer–Lemeshow hypothesis test. For Cut-off values and statistics see Table 3. *ROC* receiver operating characteristic

The need for ECMO/ECLS support as main clinical marker for reduced lung function was described with an area under the curve (AUC) of 96.59% (Fig. 4A). The Horovitz min (AUC 90.91%) and mean (AUC 74.60%) could be described by the same model, however Horovitz mean missed statistical significance (p = 0.1009).

Occurrence of AKI proofed as an inadequate parameter, as 95.7% of the patients experienced AKI. The need for dialysis, however, could be described by the same model with an AUC of 98.89% (Fig. 4B). None of the markers reached significance as predictor variables.

### CCI, sex, TLR3 in blood and IL-6 in BAL predict systemic damage

Systemic neurological damage was described by a model consisting of intercept, CCI, sex, TLR3 expression in blood and IL-6 secretion in BAL for all three clinical neurological markers of RASS mean (AUC 91.67%) need for sedation (AUC 97.22%) and mRS after 6 months (AUC 94.29%) (Table 3).

The same model also described occurrence of thromboembolic events (Fig. 4C, AUC 79.17%).

Furthermore, mortality was described with an AUC of 94.44% (Fig. 4D), showing no difference between mortality in ICU and mortality after 6 months. Again, no marker was significant as an independent predictor variable.

### CCI, sex, 1st PCT predict quality of life after 6 months

The best fitting predictive model for quality of life after 6 months showed to be intercept, CCI, sex and 1st PCT (Fig. 4E, AUC 89.42%) with the intercept (p = 0.0203) and 1st PCT (p = 0.0138) being statistically significant (Table 3).

### Patients with higher CO-Hb more likely to die

**Fig. 5:**
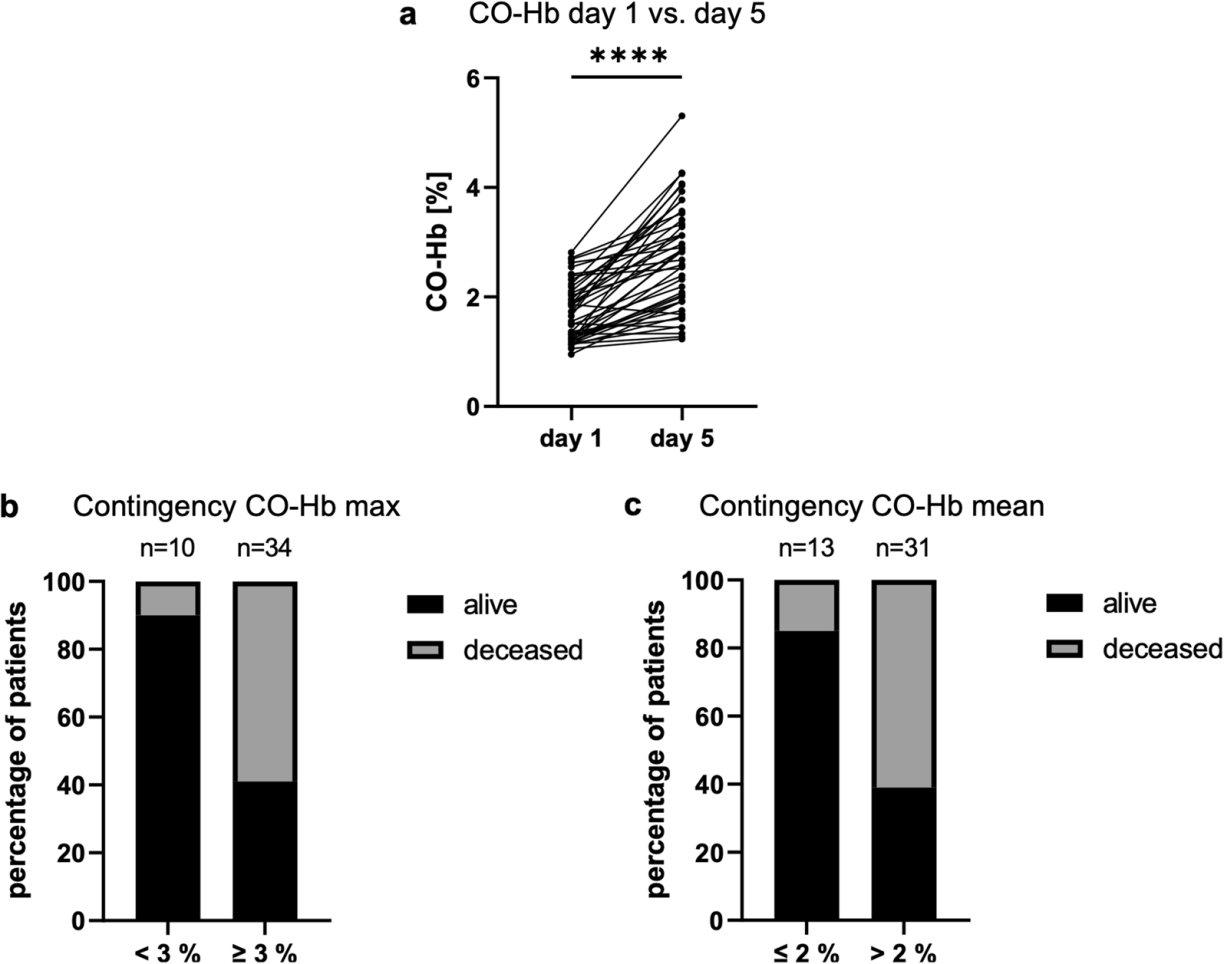
Patients with higher CO-Hb more likely to die. **a** Increase in CO-Hb values from day one to day five. Wilcoxon matched-pairs signed rank test. Contingency diagrams showing distribution of alive/deceased between patient groups of high/low **b** CO-Hb max (Fisher’s exact test: p = 0.0102, Koopman asymptotic score: RR = 2.186, 95%-CI 1.300–3.502, Baptista-Pike: OR = 12.86 95%-CI 1.947–146.9, Cut-off: 3%) and **c** CO-Hb mean (Fisher’s exact test: p = 0.0079, Koopman asymptotic score: RR = 2.186, 95%-CI 1.295–3.686, Baptista-Pike: OR = 8.708 95%-CI 1.700–42.61, Cut-off: 2%). ****p < 0.0001. *CO-Hb* carboxyhemoglobin

CO-Hb values showed a highly reproductive increase at day four to five after arrival in ICU (Fig. 5A, p < 0.0001), maintaining higher values for several days to weeks. Therefore, we excluded three patients deceased before day five from this analysis. HO-1 mRNA expression at admission did not correlate with clinical outcome. Taken together, we assumed that rather the CO-Hb-dynamic than the value at admission is of interest.

CO-Hb max (r = 0.5581, p = 0.0057) but not mean correlated to LOS ICU of survivors but neither to age.

CO-Hb mean but not CO-Hb max or CO-Hb min showed significance as predictors for mortality in ROC analyses, with CO-Hb max only just and CO-Hb min clearly missing significance (Additional file 1: Table S3).

In contingency analyses for mortality, patients with CO-Hb maximum values greater than 3% showed a relative risk (RR) of death of 2.186 (Fig. 5B) compared to patients below 3%. Likewise, patients with a mean CO-Hb higher than 2% had a RR for deceasing of 2.186 (Fig. 5C) compared to patients below 2%.

## Discussion

In this single-center prospective observational study we identified biomarkers related to inflammation and the antiviral immune response that reflect the disease course regarding organ injury and clinical outcome. Two patient characteristics are often associated with increased risk of a critical course and higher mortality in COVID-19: age [4, 5, 21] and male sex [21, 23]. In our study, age had no influence on mortality, which could however be explained by the relatively low median age of 59 years and small IQR of 11 years. We still included age into our multivariate models as part of the CCI, which accounts for comorbidities—another risk factor [4, 21, 23] and age. A higher CCI showed a non-significant trend to higher mortality and impaired QoL in survivors. As in previous studies [21], significantly more patients in our ICU were male than expected by gender distribution in society. However, within this cohort, there was no obvious sex-associated risk.

Our findings concerning general markers of inflammation are in line with previous reports. Leukocytes are known to be elevated in COVID-19 patients to healthy controls and in more severe cases [6, 10]. 1st leukocytes in our study strongly correlated with maximum and mean leukocyte counts, as well as with 1st PCT, showing a connection between inflammation at admission and during ICU treatment. 1st leukocytes mainly correlated to impaired lung function. Multivariate correlations however suggest that the first leukocytes value is no accurate predictor of disease severity.

The first PCT value on the other hand, was a good predictor of disease severity—being a significant risk factor for ECMO/ECLS and dialysis—and, in combination with sex and CCI, formed the best predictive model of QoL after 6 months. Hu et al. showed a temporary PCT increase at the beginning of COVID-19-associated hospitalization and a second increase in non-survivors until death [24]. Combined with our findings this could suggest a correlation of temporary PCT increase with disease control, while higher PCT values are associated with bacterial superinfection [25], resulting in increased lung and secondary organ injury as well as impaired QoL. However, missing influence of bacterial superinfection at admission on 1st PCT or bacterial superinfection at any time on QoL suggests a self-contained predictive value of PCT. Judgment of bacterial superinfection before admission to our ICU was hindered as many lower-level wards started calculated antibiotic therapies without attempting pathogen detection.

C-reactive protein is also often quoted as predictive factor [5, 6, 10], however it was only measured in 20 of our patients—compared to 45 patients with PCT measurements—and therefore not considered.

As TLR3 is responsible for recognizing viral RNA [9], it is surprising that we did not find an increased systemic expression compared to healthy controls. Nevertheless, increased TLR3 expression in blood showed to be a strong predictor for a less complicated course with less inflammation, less impaired lung function, better neurological short- and long-time outcome and a better QoL after 6 months. Menezes MC et al. reported a similar result; however, they were limited to TLR3 expression in blood [10]. Even though TLR3 expression in BAL was higher than in blood, it did not correlate to the clinical course. This is surprising, as a correlation between the expression in the primary spot of viral infection and the clinical outcome would seem to be likely and TLR3 induction in lung is known to help lung recovery in COVID-19 [26]. The reason for this finding needs further investigation. Potential explanations could be a discrepancy between mRNA and protein expression and TLR3 induction in the lung that was not sufficient for local virus control -hence the complicated COVID-19 course- but strong enough for systemic containment of disease.

The discrepancy in prognostic power between TLR3 expression in blood and BAL as well as the importance of blood TLR3 expression in our model for systemic damage highlights the relevance of TLR3 for a successful systemic rather than a local immunoreaction. The correlation of TLR3 expression in the blood with mRS after 14 days, at discharge and after 6 months but not at admission could hint at the relevance of systemic TLR3 for eradicating the virus during ICU treatment. However, this might be bought at the cost of thromboembolic events, as the latter could be caused by TLR3-induced endothelial dysfunction [27].

Systemic IL-8 was elevated in COVID-19 and correlated with impaired lung function and neurological impairment. However, these correlations were not significant in multivariate models, despite IL-8 blood being a significant predictor of ECMO/ECLS in ROC analyses. IL-8 in BAL was significantly higher than IL-8 in blood, which highlights the function of IL-8 to attract neutrophiles to the lungs [7, 28, 29], where they are found to be elevated in severe COVID-19 [30]. Consistently, IL-8 BAL was relevant in our model for local damage.

The correlation of IL-8 in blood and BAL with mRS at admission and systemic IL-8 with mRS after 14 days but no correlation of IL-8 with mRS at discharge or after 6 months suggests a more prominent role in the initial state of COVID-19 disease [8]. Whether IL-8 or IL-6 has better predictive relevance is still being discussed [4, 7, 8]. Our results suggest that this should be looked at with a differentiation between local and systemic damage rather than differentiating the use of IL-6 or IL-8 according to the phase of disease [8], especially as both parameters strongly correlated in blood and BAL.

Elevated IL-6 has been acknowledged as important marker for complicated courses of COVID-19 [4–6]. However, elevation of cytokine concentration in blood was shown to be less distinct than in other severe illnesses, potentially making the description as ‘cytokine storm’ exaggerated [31–33]. Our study backs the hypothesis of IL-6 measurement in BAL being more complicated but also more specific than measurement in blood [34]. IL-6 in BAL showed more correlations to clinical outcome, is part of our model for systemic damage and correlates to CO-Hb max and mean, unlike IL-6 in blood.

Correlation analyses within this study were intended to give indications about the importance of a biomarker for “local” and “systemic” damage as well as QoL. As interference with other biomarkers and demographic markers is likely, they were not intended to be used as predictive models for themselves. Likewise, ROC analyses were a further step towards developing our final three models.

Our models for local and systemic damage as well as QoL after 6 months generate useful tools to predict the most relevant clinical outcomes after assessing just four biomarkers and two easy to assess patient characteristics. For most of the questions, the models possessed an AUC > 90% and a Tjur’s R^2^ > 0.5, highlighting their accuracy. Nevertheless, there are also limitations to our models. IL-6 and IL-8 show an Odds Ratio of approximately 1 despite being often-proven risk factors and giving the models higher accuracy. Using cytokine secretion in BAL rather than in blood results in higher AUC and higher R^2^ (Additional file 1: Table S4). One theory could be, that patients for which BAL was taken shortly after admission had more critical courses and therefore secretion in BAL is likely to be different to the other patients. This argument does not exactly fit the situation in our ICU, where the regime as to when to collect a BAL changed during the study recruitment phase and was not always based on the clinical situation alone. Furthermore, even when comparing only those patients for which secretion in both, BAL and blood, was assessed, models using cytokine secretion in BAL had a higher AUC and R^2^ (Additional file 1: Table S4). Assumptions about the function of a specific marker must be taken with care, since the only significant markers are intercept and 1st PCT for QoL after 6 months. Since most of the markers, especially systemic TLR3 expression, are no part of routine laboratories in hospitals, implementation into clinical practice is not without difficulties.

Our models differ from most models published so far (for example [4, 6, 7, 11]) as our models are based on biomarker measurement after admission to ICU and therefore measurements were done at a later point in time with an already more severe disease course. We deliberately chose a different approach in a patient population already requiring maximum-level intensive care in a cross-regional center for ARDS treatment. Many of the patients showed a prolonged LOS ICU with phases of improving and phases of stagnating or even deteriorating clinical status. Medical personnel were often looking for prognostic markers to assess the likely outcome in times of missing clinical improvement. By providing such markers, our models could possibly not only help with ICU resource allocation but could also guide treatment decisions on an individual basis. While models targeting an early stage of disease and allowing for prediction of necessary ICU treatment are very helpful, our models could complement them by predicting clinical course in ICU and long-term QoL after severe COVID-19.

Within our models, outcome was categorized into “local” and “systemic” damage as well as QoL. We believe they all answer different questions and therefore recommend establishing the whole set of predictive markers for each case. This approach seems feasible as all models have several markers in common. If establishing the whole set of markers is not possible, adaptions to the most pressing clinical questions can be made. While the model for local damage could predict the need for resource intensive ECMO/ECLS and dialysis, it does also provide the markers required for predicting long-term QoL. The model for systemic damage, however, could predict the clinically important questions of potential thromboembolic events and mortality.

Considering the characteristic CO-Hb dynamic, lacking correlation between HO-1 and clinical course as well as literature findings so far [14, 35], predictive power of CO-Hb at admission seems very limited.

We could however show that the increase, which was also observed in previous studies [36, 37], was not just associated with a more complicated clinical course but could be actively used as a predictor. CO-Hb mean > 2% seems to be the more accurate predictor for death in ROC analysis than CO-Hb max $\ge$ 3%, however both strongly correlate and in contingency analysis there was no difference in RR. As it is easier to assess whether a marker exceeded a threshold at any time than to assess whether the mean measurements exceeded a threshold, use of CO-Hb max $\ge$ 3% should be considered for re-evaluating risk of death in ICU as well as indicating a prolonged stay in ICU. The increase is likely to be a sign of stress and dysregulated immune response [15]. Unlike observations in other diseases [12, 38], CO-Hb min does not seem to be a predictor in COVID-19.

There are further limitations to our study. Limiting clinical assessment to the first 14 days in ICU might improve differentiation between consequences of inflammation caused by COVID-19 and consequences of prolonged ICU-stay. However, it might also hide some clinical phenomena caused by COVID-19. Because of missing matched controls, comparisons between controls and COVID-19 patients must be taken with care. The very homogenous measurements among our controls, low age in our patient cohort and apart from TLR3 in blood missing influence of age on biomarker expression could hint at a reduced influence of age and sex on the baseline. Therefore, it is unlikely that the observed differences result from differences in age and sex alone. Since our study was a prospective single-center study at a maximum-level ICU with ECMO/ECLS therapy, patients are likely to show more severe clinical courses while not having contraindications to ECMO therapy such as high age. However, this makes our findings potentially even more important for very critical courses of COVID-19. Additionally, only 21 BALs were available at admission, which is limiting the power of our study to show differences in biomarker expression in BAL and their implications for clinical course. Since there is only a small number of cells and therefore limited mRNA in BAL, incorrect measurement in qPCR is possible. Lastly, there was no validation cohort for our prognostic models, which could lead to overestimating their predictive power. It must be pointed out that most of the correlations shown in this paper are only weak correlations (r < 0.5). Despite these limitations, there are also strengths to our study. We focused on finding biomarkers, which could differentiate between different courses of COVID-19-patients in ICU. These markers enable the medical personnel to allocate resources to the places where they are likely to be needed and to differentiate treatment. Our findings also advocate a more regular use of cytokine secretion in BAL as predictive marker and the use of CO-Hb as easy to assess parameter for re-evaluating clinical course while in ICU. Despite not finding new biomarkers, we tried to generate self-contained models explaining the mechanisms leading to multiple complications often seen at ICU in COVID-19 treatment.

## Conclusions

By using our predictive models, we were able to describe a broad range of clinically relevant short- and long-term outcomes in patients with severe COVID-19-associated ARDS. Using the described models might proof useful in risk stratification and predicting disease course in the future.

## Supplementary Information

**Additional file 1.** Supplementary methods figures and tables.

## Data Availability

The datasets used and/or analyzed during the current study are available from the corresponding author on reasonable request.

## Abbreviations

1st Leukos: First leukocyte count
1st PCT: First procalcitonin value
AKI: Acute kidney injury
ARDS: Acute respiratory distress syndrome
AUC: Area under the curve
BAL: Bronchoalveolar lavage
CCI: Charlson Comorbity Index
cDNA: Complementary desoxyribonucleic acid
CO-Hb: Carboxyhemoglobin
COVID-19: Coronavirus disease 2019
dsRNA: Double-stranded ribonucleic acid
ECMO/ECLS: Extracorporeal membrane oxygenation/extracorporeal life support
HO-1: Heme oxygenase-1
Horovitz mean: Mean Horovitz index
Horovitz min: Minimum Horovitz index
ICU: Intensive care unit
IL: Interleukin
IQR: Interquartile range
Leukos max: Maximum leukocyte count
Leukos mean: Mean leukocyte count
LOS: Length of stay
mRS: Modified Rankin Scale
NO: Nitric oxide
OR: Odds ratio
PCR: Polymerase chain reaction
PCT: Procalcitonin
Ppeak: Mean peak ventilation pressure
QoL: Quality of Life
RASS: Richmond Agitation-Sedation Scale
RNA: Ribonucleic acid
ROC: Receiver operating characteristic
Rpl13a: Ribosomal protein L13a
RR: Relative risk
SARS-CoV-2: Severe Acute Respiratory Syndrome-Coronavirus-2
TLR: Toll-like receptor
