# Supplementary material for: Predictive markers related to local and systemic inflammation in severe COVID-19-associated ARDS: a prospective single-center analysis
Source: BMC Infect Dis. 2023 Jan 11;23:19. doi: 10.1186/s12879-023-07980-z (PMC9832419; doi:10.1186/s12879-023-07980-z)
Supplement: Supplementary file 1 — Additional file 1. Supplementary methods figures and tables. [file 12879_2023_7980_MOESM1_ESM.docx]

***Additional File 1 (Supplementary Methods Figures and Tables.pdf)***

**Supplementary Methods:** Inclusion and exclusion criteria, RNA isolation and transcription into cDNA, primer sequences, Cut-off values for dichotomization of clinical outcomes

**Supplementary Figure S1:** Correlation between biomarkers

**Supplementary Table S1:** Overview of the most relevant comorbidities

**Supplementary Table S2:** Descriptive statistics of the biomarker measurements

**Supplementary Table S3:** Results of ROC analyses for ECMO/ECLS, dialysis, mortality, thromboembolic events and QoL

**Supplementary Table S4:** Comparison of Multivariate Models

**Supplementary Methods**

***Inclusion and exclusion criteria***

Inclusion criteria:

1. Adults >18 years.
2. Detection of SARS-CoV2 in naso-/oropharyngeal swab or bronchoalveolar lavage (BAL).
3. Need of ICU treatment.
4. Possibility to receive informed consent from patient, legal guardian or proxy.

Exclusion criteria:

1. Children <18 years.
2. Patients with innate immunodeficiencies.
3. Pre-existing cognitive dysfunction.
4. Death of patient within 24 hours after admission.

***RNA isolation and transcription into cDNA***

Blood samples were acquired from arterial or central venous catheters, aliquoted into two parts and stored at -80°C: One part was centrifuged and the serum collected. The other part was transferred to RNA stabilizing reagent tubes (Tempus Blood RNA Tube, AB#4342792). RNA from leucocytes was isolated via spin-column purification (Tempus Spin RNA Isolation Kit, AB#4380204) as recommended by the manufacturer.

BAL samples were centrifuged, the supernatant separated from the cell pellet and both stored at -80°C. RNA was isolated from the pellet using TRIzol^TM^ LS (Invitrogen #10296010) and concentrated by spin-column purification (RNeasy Micro Kit; Qiagen#74004).

RNA was reversely transcribed into cDNA using transcriptase polymerase chain reaction (PCR) technique (iScript cDNA Synthesis Kit, BioRad#1708891; PeqStar 96 Universal Gradient, PeqLab#732-2887).

***Primer sequences***

| TLR3 forward | GCGCTAAAAAGTGAAGAACTGGAT |
| --- | --- |
| TLR3 reverse | GCTGGACATTGTTCAGAAAGAGG |
| HO-1 forward | GTGATAGAAGAGGCCAAGACTG |
| HO-1 reverse | GAATCTTGCACTTTGTTGCTGG |
| Rpl13a forward | CGGACCGTGCGAGGTAT |
| Rpl13a reverse | CACCATCCGCTTTTTCTTGTC |

***Cut-off values for dichotomization of clinical outcomes***

- Horovitz min >72 mmHg [favorable] vs. <72 mmHg [unfavorable]
- Horovitz mean >150 mmHg [favorable] vs. <150 mmHg [unfavorable]
- ECMO/ECLS support yes/no
- dialysis yes/no
- RASS mean >-4 [favorable] vs. <-4 [unfavorable]
- need for sedation <0.5 [favorable] vs. >0.5 [unfavorable]
- mRS after six months 0-2 [favorable] vs. 3-6 [unfavorable]
- thromboembolic events yes/no
- mortality yes/no
- EQ-5D-5L index >0.7 [favorable] vs. <0.7 [unfavorable]

**Supplementary Figure S1: *Correlation between biomarkers***


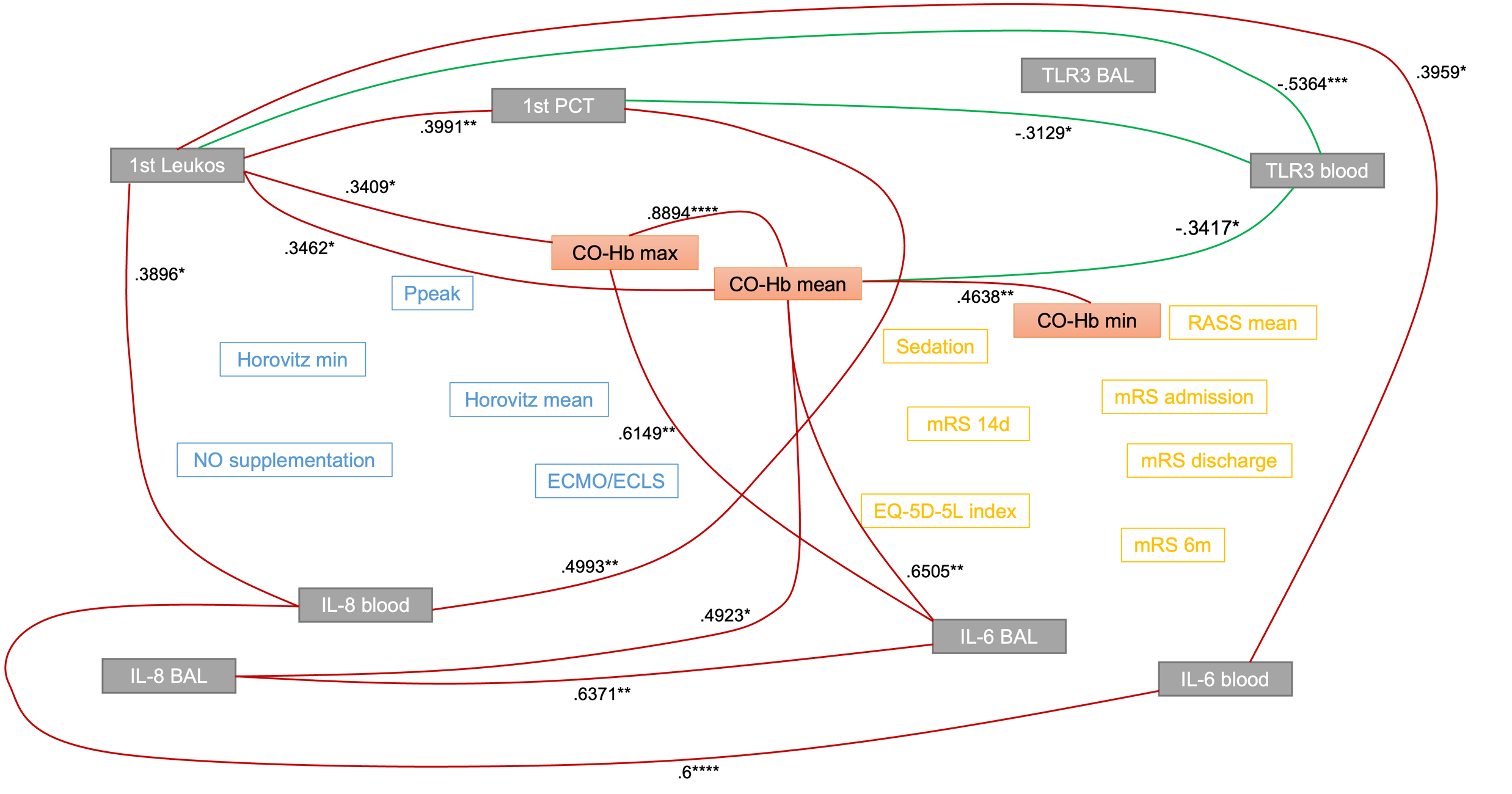


Red arrow, positive correlation, green arrow, negative correlation. *p<0.05, **p<0.01, ***p<0.001, ****p<0.0001. *PCT, procalcitonin, CO-Hb, carboxyhemoglobin, BAL, bronchoalveolar lavage*

In blood, 1^st^ Leukos correlated to all biomarkers, 1^st^ PCT to all except IL-6. Systemic TLR3 negatively correlated to 1^st^ Leukos and 1^st^ PCT but to no other biomarker. Systemic IL-6 and IL-8 showed strong positive correlation (r=0.6000; p<0.0001). When compared to blood, BAL showed significantly higher expression of TLR3 (p<0.0001) and secretion of IL-8 (p=0.0135), while IL-6 secretion in BAL showed no difference to the secretion in blood. In BAL only IL-6 and IL-8 correlated (r=0.6371; p=0.0033).

Apart from positive correlations to 1^st^ Leukos, CO-Hb max and mean were positively correlated to IL-6 secretion in BAL as well as with each other (r=0.8894; p<0.0001). CO-Hb mean was also negatively correlated to systemic TLR3 and positively correlated to IL-8 secretion in BAL and CO-Hb min.

**Supplementary Table S1: *Overview of the most relevant comorbidities***

| No. of patients |  |  |
| --- | --- | --- |
| **Part of CCI** | cerebrovascular disease | 1 |
|  | chronic lung disease | 7 |
|  | collagenosis | 3 |
|  | gastroduodenal ulcus disease | 4 |
|  | Mild liver disease | 3 |
|  | Diabetes mellitus | 9 |
|  | Hemiplegia/cerebrovascular event | 4 |
|  | Moderate/severe liver disease | 3 |
|  | Tumor without metastases | 1 |
|  | Lymphoma | 2 |
|  | Bronchial asthma | 3 |
|  | Did ever smoke | 8 |
|  | Arterial hypertension | 30 |
|  | BMI >35 | 9 |
|  | Surgery within 6 months before admission | 3 |
|  | Psoriasis | 3 |
|  | Benign prostate hyperplasia | 4 |
|  | Thyroid disease of any kind | 5 |
|  | Ophthalmologic disease of any kind | 4 |
|  | Cardiovascular disease of any kind (e.g. coronary heart disease, atrial fibrillation, anemia) | 8 |
|  | Gastrointestinal disease of any kind (e.g. diverticulitis, gastroesophageal reflux disease, chronic pancreatitis) | 6 |
|  | Neurological/mental disease of any kind (e.g. M. Parkinson, epilepsy, depression) | 8 |

Summary of the most relevant comorbidities. Chronic lung diseases and bronchial asthma did not significantly influence biomarker expression in BAL, tumors and lymphoma did not significantly influence biomarker expression in blood (all p>0.05 in ROC analyses). *No., number, CCI, Charlson Comorbidity Index, BMI, body mass index, e.g., for example.*

**Supplementary Table S2: *Descriptive statistics of the biomarker measurements***

| **a) Biomarker measurements in blood** | | | | | | | | |
| --- | --- | --- | --- | --- | --- | --- | --- | --- |
|  | Number of values | 25% Percentile | Median | 75% Percentile | IQR | Mean | Std. Deviation | Std. Error of Mean |
| TLR3 COVID-19 | 44 | -0.867 | -0.06613 | 0.9789 | 1.8459 | -0.1178 | 1.473 | 0.2221 |
| IL-6 COVID-19 [pg/ml] | 40 | 150.4 | 350.5 | 705.7 | 555.3 | 582.9 | 711.8 | 112.5 |
| IL-8 COVID-19 [pg/ml] | 40 | 65.42 | 122.2 | 179.1 | 113.68 | 177.7 | 196.2 | 31.03 |
| HO-1 COVID-19 | 44 | 1.238 | 1.784 | 2.147 | 0.909 | 1.755 | 0.7587 | 0.1144 |
| TLR3 controls | 5 | -0.554 | 0.2612 | 0.4234 | 0.9774 | 0.000 | 0.5358 | 0.2396 |
| IL-6 controls [pg/ml] | 4 | 0 | 0 | 0 | 0 | 0 | 0 | 0 |
| IL-8 controls [pg/ml] | 4 | 4.465 | 12.55 | 16.18 | 11.715 | 11.06 | 6.346 | 3.173 |
| HO-1 controls | 5 | -0.3144 | -0.1179 | 0.3734 | 0.6878 | 0 | 0.4345 | 0.1943 |
| 1st Leukos [10^3^/μl] | 47 | 8.53 | 11.35 | 15.21 | 6.68 | 12.87 | 6.628 | 0.9668 |
| 1st PCT [ng/ml] | 45 | 0.265 | 0.87 | 4.79 | 4.525 | 3.927 | 6.186 | 0.9221 |
| CO-Hb max [%] | 44 | 3 | 3.8 | 4.575 | 1.575 | 3.809 | 1.079 | 0.1626 |
| CO-Hb mean [%] | 44 | 1.935 | 2.47 | 3.118 | 1.183 | 2.595 | 0.8088 | 0.1219 |
| CO-Hb min [%] | 44 | 0.8 | 1 | 1.275 | 0.475 | 1.039 | 0.4484 | 0.06759 |
| **b) Comparisons between measurements in blood and in BAL** | | | | | | | | |
|  | Number of values | 25% Percentile | Median | 75% Percentile | IQR | Mean | Std. Deviation | Std. Error of Mean |
| TLR3 blood | 44 | -12.92 | -12.12 | -11.08 | 1.84 | -12.18 | 1.473 | 0.2221 |
| IL-6 blood [pg/ml] | 40 | 150.4 | 350.5 | 705.7 | 555.3 | 582.9 | 711.8 | 112.5 |
| IL-8 blood [pg/ml] | 40 | 65.42 | 122.2 | 179.1 | 113.68 | 177.7 | 196.2 | 31.03 |
| HO-1 blood | 44 | -4,541 | -3,995 | -3,632 | 0,909 | -4,024 | 0,7587 | 0,1144 |
| TLR3 BAL | 21 | -11.21 | -10.14 | -9.009 | 2.201 | -10.11 | 1.168 | 0.2549 |
| IL-6 BAL [pg/ml] | 19 | 28.06 | 487.8 | 1258 | 1229.9 | 1036 | 1773 | 406.7 |
| IL-8 BAL [pg/ml] | 19 | 61.81 | 515.1 | 4519 | 4457.2 | 3022 | 4736 | 1086 |
| HO-1 BAL | 21 | -4,109 | -3,403 | -2,363 | 1,746 | -3,34 | 1,522 | 0,3322 |

Descriptive statistics on biomarker measurements **a)** in blood and **b)** for comparisons between blood and BAL. Differing values for TLR3 and HO-1 measurements in blood are caused by the use of the –ΔCT value for comparing blood and BAL instead of the –ΔΔCT value used for comparing patients and controls. This was necessary as there were no control BAL samples.

|  | ECMO/ECLS | | Dialysis | | Thromboembolic events | | Mortality | | QoL | |
| --- | --- | --- | --- | --- | --- | --- | --- | --- | --- | --- |
|  | AUC | p-value | AUC | p-value | AUC | p-value | AUC | p-value | AUC | p-value |
| 1st Leukos | **0.6938** | **0.0228** | 0.5109 | 0.8982 | 0.6091 | 0.2008 | 0.5652 | 0.4436 | 0.5357 | 0.7848 |
| 1st PCT | **0.7143** | **0.0140** | **0.6825** | **0.0363** | 0.5257 | 0.7679 | 0.5484 | 0.5780 | 0.7356 | 0.0760 |
| TLR3 blood | **0.7314** | **0.0086** | 0.5579 | 0.5110 | 0.5620 | 0.4813 | 0.6438 | 0.1039 | 0.6538 | 0.2926 |
| IL-6 blood | 0.6535 | 0.1007 | 0.5518 | 0.5773 | 0.6025 | 0.2674 | 0.5038 | 0.9675 | 0.5288 | 0.8280 |
| IL-8 blood | **0.7136** | **0.0223** | 0.5669 | 0.4713 | 0.5888 | 0.3369 | 0.5808 | 0.3843 | 0.7452 | 0.0648 |
| IL-6 BAL | 0.5852 | 0.5357 | 0.6056 | 0.4379 | 0.6556 | 0.2530 | 0.6444 | 0.2885 | 0.5952 | 0.6485 |
| IL-8 BAL | 0.5568 | 0.6797 | 0.5000 | >0.9999 | 0.5667 | 0.6242 | 0.6111 | 0.4142 | 0.6190 | 0.5688 |
| CO-Hb max | N/A | N/A | N/A | N/A | N/A | N/A | 0.6677 | 0.0570 | N/A | N/A |
| CO-Hb mean | N/A | N/A | N/A | N/A | N/A | N/A | **0.7070** | **0.0188** | N/A | N/A |
| CO-Hb min | N/A | N/A | N/A | N/A | N/A | N/A | 0.5072 | 0.9345 | N/A | N/A |

**Supplementary Table S3: *Results of ROC analyses for ECMO/ECLS, dialysis, mortality, thromboembolic events and QoL***

Presented are the area under the curve (AUC) and corresponding p-values. Bold text signaling significant results, *N/A, not tested. ROC, receiver operating characteristic, QoL, quality of life after 6 months*

We analyzed the ability of each parameter to predict the five main clinical outcomes, i.e., ECMO/ECLS, dialysis, mortality, thromboembolic events and QoL via ROC analysis. 1^st^ PCT, 1^st^ Leukos, TLR3 blood and IL-8 blood were significant predictors for ECMO/ECLS. The biomarkers discussed so far missed further significance in all other ROC analyses apart from 1^st^ PCT as predictor for dialysis. Systemic IL-6, IL-6 BAL and IL-8 BAL missed any significance.

**Supplementary Table S4: *Comparison of Multivariate Models***

|  | Cytokine BAL | | Cytokine blood | | Cytokine blood same patients as BAL | |
| --- | --- | --- | --- | --- | --- | --- |
|  | AUC | R^2^ | AUC | R^2^ | AUC | R^2^ |
| Horovitz min | 0.9091 | 0.5754 | 0.8663 | 0.2646 | 0.9091 | 0.5939 |
| Horovitz mean | 0.7460 | 0.2456 | 0.7867 | 0.1863 | 0.7460 | 0.1247 |
| ECMO/ECLS | 0.9659 | 0.7312 | 0.8152 | 0.2966 | 0.8977 | 0.4598 |
| Dialysis | 0.9889 | 0.8074 | 0.8610 | 0.1732 | Cannot fit regression | |
| RASS mean | 0.9167 | 0.5197 | 0.8061 | 0.2475 | 0.9167 | 0.5124 |
| Sedation | 0.9722 | 0.7544 | 0.7690 | 0.2202 | 0.9306 | 0.5175 |
| mRS 6m | 0.9429 | 0.6199 | 0.8833 | 0.4323 | 0.9571 | 0.7408 |
| Thromboembolic events | 0.7917 | 0.2705 | 0.6053 | 0.03468 | 0.6806 | 0.1117 |
| Mortality | 0.9444 | 0.5711 | 0.7544 | 0.1825 | 0.9444 | 0.5987 |

Multivariate models using cytokine secretion in BAL more accurate than models using cytokine secretion in blood. Presented are AUC and Tjur’s R2. *AUC, area under the curve*
